# 

**Published:** 1820-01-01

**Authors:** 


### *Additional Patronage since last Quarter.*

Allan, Mr. J. Surgeon, No. 1, Jermyn-street, St. James's
Arnott, Dr. Bedford Square
Bell, Mr. William, Assistant-Surgeon, Trincomalee Hospital, Ceylon
Bell, Mr. Alexander, Member of the Royal College of Surgeons, London, Surgeon to the Dundee Royal Infirmary
Boutflower, Charles, Esq. Surgeon to the Forces, Colchester
Bree, Dr. Robert, George-street, Hanover-square
Breschet, M. M. Chef des Travaux Anatomiques, Hospice de Perfectionment a Paris
Chapman, Mr. Surgeon, Windsor
Clarke, Dr. John Cutbert, Smyrna
Coley, J. M. Esq. Bridgnorth
Colquhoun, Dr. Craven-street, Strand
Cornish, Mr. James, Falmouth
Crichton, Dr. West Indies
Dawson, Ensign, St. Kitts, West Indies
Delph, Mr. Surgeon, Northumberland Place, Edmonton
Dobie, Mr. Exmouth-street, Spa Fields
Draper, Mr. Surgeon, Burford, Oxfordshire
Dwyer, Dr. John, Physician to the Forces, Ceylon
Emerson, Dr. A. L. Brompton
Fitzpatrick, Mr. Royal Artillery, Woolwich
Fewster, Mr. Thomas, Surgeon, Thornbury, Gloucestershire
Forbes, Dr. Argyle-street
Forman, Mr. George Ellery, Member of the Royal College of Surgeons, London
Fothergill, Dr. William, late Editor of the Medical and Physical Journal, Jamaica
Grant, Mr. Surgeon, Bristol
Hamet, Dr. East-Indies, with the Commander in Chief
Harold Philip, Esq. Surgeon, Holt, Norfolk
Hassel, Dr. and Surgeon, Boulogne Sur-Mer
Hume, Dr.
Hunter, Dr. Richmond
Jackson, Dr. Jamaica
Jardine, W. Esq. Surgeon, R. N. Dumfries
Jenner, Dr. Edward, Berkeley, Gloucestershire
Lewis, George, Esq. Surgeon, Sudbury
Macewan, Mr. George, Surgeon, Grenada
Morel, Mr. New Bond Street
Moon, Mr. Medical Staff
Park, Mr. Surgeon, Kingsland Road
Penrith Medical Society, Cornwall
Porter, Dr. Arthur, Little Hanover, New Hampshire
Powell, W. H. Esq. Surgeon, Manchester-street, Manchester-square
Reay, Mr. Surgeon
Rodd, Mr. Surgeon, Hampstead
Rouzet, Dr. Rue des St Peres, a Paris, in Exchange for "La Revue Medicale, &c."
Rudland, W. H. Esq. Surgeon, Royal Navy, Dartmouth
Sawyer, Mr. Margate
Scatchard, Mr. John, Surgeon, East Keswick, near Leeds
Schoolbred, Mr. London Hospital
Scott, Dr. John
Shipton, Mr. Chipping
Stowe, Dr. William, Buckingham
Thomson, Dr. Conduit-street
Thompson, Mr. Merchant, West Indies
Travis and Dunn, Messrs. Scarbro'
Trevosso, Mr. Surgeon, Falmouth
Vaile, William Phelps, Esq. Member of the Royal College of Surgeons, London, Headcorn, Kent.
Wadd, William, Esq. Park-place, St. James's
Weatherhead, Dr. Montague-street, Montague-square
Williamson, Mr. Whitehaven
Wilmore, E. W. Esq. Worcester
Young, Dr. James Forbes, Paradise Row, Lambeth

---

*** The next Number will close the Second Volume, and exhibit a General List of Subscribers who have forwarded their Names. Let it be always understood, however, that Subscribers are to procure and pay for the Journal through their own Booksellers, the same as for any other Publication.

